# Motor development related to duration of exclusive breastfeeding, B vitamin status and B12 supplementation in infants with a birth weight between 2000-3000 g, results from a randomized intervention trial

**DOI:** 10.1186/s12887-015-0533-2

**Published:** 2015-12-18

**Authors:** Ingrid Kristin Torsvik, Per Magne Ueland, Trond Markestad, Øivind Midttun, Anne-Lise Bjørke Monsen

**Affiliations:** Department of Pediatrics, Haukeland University Hospital, N-5021 Bergen, Norway; Laboratory of Clinical Biochemistry, Haukeland University Hospital, N-5021 Bergen, Norway; Institute of Medicine, Faculty of Medicine and Dentistry, University of Bergen, N-5021 Bergen, Norway; Department of Clinical Science, Faculty of Medicine and Dentistry, University of Bergen, N-5021 Bergen, Norway; Bevital AS, N-5021 Bergen, Norway

**Keywords:** B vitamins, cobalamin, motor development, infants, breastfeeding

## Abstract

**Background:**

Exclusive breastfeeding for 6 months is assumed to ensure adequate micronutrients for term infants. Our objective was to investigate the effects of prolonged breastfeeding on B vitamin status and neurodevelopment in 80 infants with subnormal birth weights (2000-3000 g) and examine if cobalamin supplementation may benefit motor function in infants who developed biochemical signs of impaired cobalamin function (total homocysteine (tHcy) > 6.5 μmol/L) at 6 months.

**Methods:**

Levels of cobalamin, folate, riboflavin and pyridoxal 5´-phosphate, and the metabolic markers tHcy and methylmalonic acid (MMA), were determined at 6 weeks, 4 and 6 months (*n* = 80/68/66). Neurodevelopment was assessed with the Alberta Infants Motor Scale (AIMS) and the parental questionnaire Ages and Stages (ASQ) at 6 months.

At 6 months, 32 of 36 infants with tHcy > 6.5 μmol/L were enrolled in a double blind randomized controlled trial to receive 400 μg hydroxycobalamin intramuscularly (*n* = 16) or sham injection (*n* = 16). Biochemical status and neurodevelopment were evaluated after one month.

**Results:**

Except for folate, infants who were exclusively breastfed for >1 month had lower B vitamin levels at all assessments and higher tHcy and MMA levels at 4 and 6 months. At 6 months, these infants had lower AIMS scores (*p* = 0.03) and ASQ gross motor scores (*p* = 0.01).

Compared to the placebo group, cobalamin treatment resulted in a decrease in plasma tHcy (*p* < 0.001) and MMA (*p* = 0.001) levels and a larger increase in AIMS (p = 0.02) and ASQ gross motor scores (*p* = 0.03).

**Conclusions:**

The findings suggest that prolonged exclusive breastfeeding may not provide sufficient B vitamins for small infants, and that this may have a negative effect on early gross motor development. In infants with mild cobalamin deficiency at 6 months, cobalamin treatment significantly improvement cobalamin status and motor function, suggesting that the observed impairment in motor function associated with long-term exclusive breastfeeding, may be due to cobalamin deficiency.

**Clinical trial registration:**

ClinicalTrials.gov, number NCT01201005

## Background

Infant micronutrient status depends on gestational age (GA), birth weight (BW), and maternal micronutrient status during pregnancy and after delivery for infants who are breastfed [1, 2]. For infants born at term with an appropriate weight for GA (AGA), exclusive breastfeeding is believed to ensure an adequate supply of micronutrients during the first 6 months [3], whereas iron, folic acid or multivitamin supplementations are usually given to infants with a BW below 2500 g (g) [4, 5]. Breast milk is important for the infant, but it is however, not a complete food, as it is low in vitamins K and D [6, 7]. Vitamin K injections to neonates and a minimum daily intake of 400 IU (10 μg) of vitamin D beginning soon after birth are therefore recommended by many countries [8–10]. There have also been concerns about low levels of other vitamins in breast milk, namely vitamin A, vitamin B2 (riboflavin), vitamin B6 and vitamin B12 (cobalamin) [1, 11, 12], but routine supplementation of these vitamins to breastfed infants of under-nourished mothers has not been implemented [1, 13].

As formula is supplemented with several B vitamins, deficiency is uncommon in formulafed infants [14, 15]. Folate levels are reported to be high in breast milk, and folate deficiency in term born AGA breastfed infants is uncommon [16]. There are few data on the prevalence of vitamin B2 and B6 deficiency among young infants, but studies in both low-income and high-income countries have documented a rather high incidence of deficiency of both vitamins among pregnant and lactating women [17, 18]. Total cobalamin concentration in human milk falls progressively during the lactation period [12, 19], and in exclusively breastfed term infants with an adequate birth weight, a biochemical profile indicative of impaired vitamin B12 status has been reported to be common from 4 months [12, 20]

An adequate micronutrient status is important to support optimal growth and development during infancy [21]. In a recent intervention study, cobalamin supplementation resulted in biochemical evidence of cobalamin repletion and improvement in motor function and regurgitations in term infants up to the age of 8 months, demonstrating that an adequate cobalamin status is important for a rapidly developing nervous system [22]. Other micronutrients, including iron and zinc, have also been shown to play an important role in infant motor development [23].

Low BW is a known risk factor for both developmental delays and lower stores of several micronutrients [24], which in turn may affect gross motor development [25, 26]. We investigated B vitamin status during the first 6 months of life in infants with a subnormal BW (2000-3000 g), in relation to nutrition, i.e. exclusive breastfeeding for 0–1 month or ≥ 1 month. The association between gross motor development, nutrition and B vitamin status was assessed at 6 months. Infants with biochemical signs of cobalamin deficiency at 6 months were included in a randomized cobalamin intervention study, and biochemical status and motor development were evaluated after one month.

## Methods

### Study population and design

Between December 2008 and April 2010, 97 healthy infants with a BW 2000-3000 g and their mothers were consecutively recruited at the Department of Obstetrics and Gynecology, Haukeland University Hospital, Bergen, Norway. Determination of gestational age (GA) was based on ultrasonography at 17–18 weeks’ gestation and small for gestational age (SGA) was defined as BW less than the 10^th^ percentile for GA according to recently updated growth charts for Norwegian infants [27].

The infants and their mothers were invited back for investigation at 6 weeks, 4 months and 6 months. At each visit the infants’ growth parameters were measured, a questionnaire on infant and maternal nutrition and vitamin supplementation was completed and blood samples were collected from the infant and the mother. At 6 months, infant neurodevelopment was assessed. In infants, cobalamin is the main determinant of plasma tHcy [2, 28] and a plasma tHcy level of 6.5 μmol/L was chosen as a cut-off for defining impaired cobalamin function [29]. Infants with a tHcy level >6.5 μmol/L at 6 months were invited to a double blind randomized controlled cobalamin intervention study, and biochemical status and motor development were evaluated after one month.

All infants received sugar water for pain relief during blood sampling and during injection for those included in the intervention study [30]. The Regional Committee for Medical and Health Research Ethics West granted ethical approval of the protocol, and the mothers gave written, informed consent. An additional written, informed consent was given by the mothers included in the intervention trial. The trial is registered with ClinicalTrials.gov, number NCT0 1201005.

### Nutrition

According to Norwegian recommendations all infants receive vitamin D (10 μg per day) as cod liver oil or vitamin D drops from 6 weeks of age [31]. Infants with a BW ≤ 2500 g also receive a multivitamin supplement for the first 3 weeks after being discharged from the hospital, iron supplements from 6 weeks to 1 year and folic acid from 3 days to 3 months of age. In this study multivitamins were provided as Multibionta, (Merck Selbstmedikation GmbH, Darmstadt, Germany), iron as ferrous fumarate mixture, (Nycomed Pharma AS, Asker, Norway), 9 mg daily from 6 weeks to 6 months, and 18 mg daily to 12 months of age, and folic acid (Apotek, Oslo, Norway), 0.1 mg daily.

Infant nutrition was recorded as exclusive breastfeeding or mixed feeding, which included breastfeeding combined with infant formula, exclusive infant formula feeding or either of these combined with cereals or solid foods. Infants who were never breastfed or exclusively breastfed for less than 1 month were categorized as formula fed and infants who were exclusively breastfed for more than 1 month were categorized as breastfed. Months of breastfeeding was also used as a continuous variable. It was recommended that solid food, usually starting with infant cereals, was introduced at 6 months of age. The different cereals contained 3–10 mg iron, 15–45 μg folic acid and 0.09–0.3 mg vitamin B6 per 100 g powder. The various formulas contained 0.41–1.22 mg iron, 0.06–0.16 mg riboflavin , 0.02–0.05 mg vitamin B6 , 0.09–0.24 μg cobalamin and 6–15 μg folic acid per 100 ml prepared milk.

The official guideline in Norway is to take a daily folic acid supplement of 0.4 mg from 1 month before and throughout the first 2–3 months of pregnancy; however, only 10% follow this recommendation [32]. Approximately 80 % of the folic acid users report taking an additional micronutrient supplements during the first trimester [33].

### Neurodevelopmental assessment

At 6 months the infants underwent a pediatric examination and neurodevelopmental evaluation by one pediatrician (IT), using the Alberta Infants Motor Scale (AIMS) test [34] and the parental questionnaire Ages and Stages Questionnaire (ASQ) [35].

#### AIMS

This is a norm-referenced observational tool designed for evaluating gross motor development in infants from birth to 18 months [36]. Assessment is based on free observation of the child in different positions (prone, supine, sitting and standing) according to the age of the child. The obtained score, 0 to 60 points, is converted to a normative age-dependent percentile rank (5th to 90th percentile). A score below the 10^th^ percentile is classified as possibly delayed motor development [36].

All infants were videotaped during the AIMS test. All scores were revised based on the videotapes, without access to clinical data, after the study was completed. The AIMS test was not possible to obtain for all infants (missing *n* = 5), because the infant was sleepy or distressed.

#### ASQ

To assess neurodevelopment, the Norwegian version of the 6-month form of ASQ was used. This is a validated parent-completed developmental screening tool with a high sensitivity and specificity to detect developmental delay [37, 38]. ASQ covers 5 developmental domains, i.e. communication, gross motor function, fine motor function, personal-social functioning and problem solving, and each domain has 6 questions on the developmental milestones. The parents evaluate whether the child has achieved a milestone (yes, 10 points), has partly achieved the milestone (sometimes, 5 points) or has not yet achieved the milestone (no, 0 points). Sums of each domain scores were calculated for every infant.

### Cobalamin intervention

At 6 months, infants with impaired cobalamin function (tHcy level >6.5 μmol/L) were invited to participate in an intervention study. Eligible infants were assigned by block randomization (envelopes, 10/10) to receive either an intramuscular injection of 400 μg hydroxycobalamin (Vitamin B12 Depot, Nycomed Pharma, Norway) (cobalamin group, *n* = 16), or a sham injection, i.e. the skin was punctured by a needle connected to a syringe (placebo group, *n* = 16). These procedures were performed by one pediatrician (ALBM), and the parents were blinded to whether their infant received cobalamin or not (both syringes were wrapped in aluminium foil in order to hide the content, and the parent was asked to turn her head away, to prevent her from observing whether the syringe was activated). Assignment to cobalamin and placebo group was also blinded to the pediatrician (IT) who performed all the clinical and developmental assessments, and to the laboratory personnel. All infants were scheduled for follow-up one month after the first examination and this included blood tests, AIMS evaluation (IT) and maternal questionnaire concerning nutrition, growth and ASQ.

### Blood sampling and analyses

Blood samples from the infants and the mothers were obtained by antecubital venipuncture and collected into EDTA Vacutainer Tubes (Becton Dickinson) for separation of plasma and in Vacutainer Tubes without additives (Becton Dickinson) for separation of serum. Blood samples for preparation of EDTA-plasma were placed in ice water, and plasma was separated within 4 h. The samples were stored at –80 °C until analysis. Plasma levels of total homocysteine (tHcy) and methylmalonic acid (MMA) were assayed using a (GC-MS) method based on methylchloroformate derivatization [39]. Serum cobalamin was determined by a Lactobacillus leichmannii microbiological assay [40], serum folate by a Lactobacillus casei microbiological assay [41] whereas plasma levels of riboflavin and pyridoxal 5´-phosphate (PLP, the active form of vitamin B6) were analyzed using an LC-MS/MS assay [42]. A complete set of vitamin and metabolites was not available for all infants at all time points. Analyses of vitamins and biomarkers were carried out at BEVITAL AS (www.bevital.no).

### Statistical analysis

Results are presented as median and interquartile range (IQR) and mean and standard deviation. Medians were compared by Mann-Whitney U test, and means with Student’s t-test. Differences in categorical variables were tested with the Chi-square test.

Multiple linear regression models were used to assess the relation of AIMS scores at 6 months with gender, SGA, weight at 6 months, folic acid and iron supplementation, number of months with exclusive breastfeeding and maternal education.

Graphical illustration of the dose-response relationship between months of exclusive breastfeeding versus concentrations of cobalamin, folate, PLP, riboflavin, tHcy and MMA levels at 6 months and between AIMS score and tHcy and MMA levels at 6 months were obtained by generalized additive models (GAM). The models were adjusted for folic acid and iron supplementation (i.e. for infants with BW ≤ 2500 g).

The calculation of the sample size for the intervention study was based on data from our previous cobalamin intervention study in infants below 8 months [22]. A calculated sample size of 36; i.e. 18 in each group, would give the study a statistical power of more than 80 % to detect a 1.9 difference in AIMS increment score at a 5 % significance level.

GAMs were computed using the mgcv-package (version 1.4–1) in R (The R Foundation for Statistical Computing, version 2.8.1), and the SPSS statistical package (version 18) was used for the remaining statistical analyses. Two-sided p-values < 0.05 were considered statistically significant.

## Results

### Demographics and Nutrition

#### Infants

Of the 97 infant-mother dyads initially recruited at birth, 80 infants (including 8 pairs of twins and 1 single twin) returned at 6 weeks, and were included in either the formula fed group (*n* = 32, 40 %) or the breastfed group (*n* = 48, 48 %). The formula fed group comprised infants who were never breastfed (*n* = 27) and infants who were exclusively breastfed for less than 1 month (*n* = 5), whereas the breastfed group included infants who were exclusively breastfed for more than 1 month. Mean GA was 37 weeks (SD 1.8), 41 % were premature, and 33 % were SGA. Apart from a higher percentage of twins in the formula fed group, there were no differences in infant characteristics between the formula fed and breastfed infants (Table 1).

**Table 1:** Characteristics of infants and mothers, growth and neurodevelopmental assessment according to nutrition

	Duration of exclusive breastfeeding (Group)		*P* ^a^
Characteristics of infants	0–1 month (Formula fed)	>1 month (Breastfed)	
Number at inclusion	32	48	
Number at 6 months	26	40	
Gender (M) [*n* (%)]	13 (50)	20 (50)	1
Birth weight (g)	2458 ± 294^b^	2561 ± 224	0,12
Gestational age (weeks)	36.9 (1.9)	37.3 (1.8)	0,42
Premature [*n* (%)]	10 (39)	16 (40)	0,90
SGA [*n* (%)]	7 (30)	13 (33)	0,63
Twins [*n* (%)]	10 (39)	4 (10)	0,006
Exclusive breastfeed (months)	0 (0)^c^	5 (3.4, 5.4)	0,02
Folate and iron supplementation [*n* (%)]^d^	16 (62)	14 (35)	0,03
Multivitamin supplementation [*n* (%)]^e^	11 (42)	12 (30)	0,31
Characteristics of mothers			
BMI prior to pregnancy (kg/m2)	23.7 (4.0)	22.5 (3.3)	0.19
Higher education [*n* (%)]^f^	10 (42)	28 (70)	0,03
Plasma MMA μmol/l at 6 months	0.15 (0.13–0.18)	0.18 (0.16–0.21)	0.01
Plasma tHcy μmol/l at 6 months	7.17 (5.91–9.69)	7.86 (7.05–10.95)	0.10
Growth and neurodevelopment at 6 month			
Weight (g)	7256 ± 646	7019 ± 894	0,25
Weight gain (g)^g^	4797 ± 750	4458 ± 907	0,10
AIMS (score)	24 (22, 27)	21 (18, 25)	0,03
AIMS (percentile)	50–75 (25–50, 75)	25–50 (25, 50)	0,01
ASQ, communication (score)	48 (40, 50)	45 (35, 50)	0.35
ASQ, gross motor (score)	40 (35, 49)	35 (25, 40)	0.01
ASQ, fine motor (score)	50 (36, 60)	35 (30, 50)	0.06
ASQ, problem solving (score)	50 (50, 60)	50 (40, 58)	0.22
ASQ, personal-social (score)	45 (35, 50)	45 (35, 53)	0.66

^*a*^Proportions were compared by chi-square test. Means were compared by student’s t-test. Medians were compared by mann-Whitney U test

^*b*^Mean ± SD (all such values)

^*c*^Median; IQRs in parentheses (variable that was not normally distributed) (all such values)

^*d*^Folic acid supplementation 0.1 mg daily from day 3 to 3 months

^*e*^Multivitamin supplementation the first 3 weeks of life

^*f*^Minimum 3 years of college or university education (one missing in each group)

^*g*^Weight gain from birth to 6 months

*SGA* Small for gestational age < 10percentila, *AIMS* Alberta Infant Motor Scale, AIMS was missing for 5 infants, *ASQ* Ages and stages questionnaires, ASQ was missing for 5 infants

At 4 months, 12 infants were lost to follow-up (8 from the breastfed group and 4 from the formula fed group) and at 6 months additional 2 infants were lost to follow-up in the formula fed group. These 14 infants showed no significant differences in baseline characteristics compared to the study group at 6 weeks (all *p* > 0.21).

As recommended, all infants received cod liver oil or other vitamin D supplementation from age 6 weeks and infants with BW ≤ 2500 g (*n* = 36, 45 %) also received iron (100 %), folic acid (100 %) and multivitamin supplement (78 %).

#### Mothers

A higher proportion of the breastfeeding mothers had higher education and they tended to have a lower pre pregnancy body mass index (Table 1). Age, parity and number of previous pregnancies were the same for the groups.

Daily use of multivitamin supplement for a shorter or longer period was reported by 38 % of the mothers during pregnancy, and by 28 % postpartum up to 6 months, with no significant differences between the groups (*p* > 0.29). Apart from a higher MMA level at 6 months in the breastfeeding compared to the formula feeding mothers (Table 1), no significant differences were observed in maternal B vitamin status between the two groups (*p* > 0.10). During follow-up, the mothers had a fairly stable vitamin B status except for PLP, which increased from 6 weeks to 6 months. Maternal PLP and riboflavin levels were considerably lower than in the infants.

### Infant vitamin status in relation to breastfeeding practice

At 6 months, duration of exclusive breastfeeding in months from birth was inversely associated with infant B vitamin levels, i.e. cobalamin (r = -0.55, *p* < 0.001), PLP (r = -0.53, *p* < 0.001), riboflavin (r = -0.57, *p* < 0.001), and positively associated with the metabolic markers, tHcy (r = 0.47, *p* < 0.001) and MMA (r = 0.55, *p* < 0.001). No association was observed between duration of exclusive breastfeeding and folate level (r =0.01, *p* = 0.97).

Although cobalamin, PLP and riboflavin levels increased somewhat in the breastfed infants from 6 weeks to 6 months, the formula fed infants had at all assessments significantly higher levels of these vitamins and at 4 and 6 months also significantly lower levels of the metabolic markers tHcy and MMA compared to breastfed infants (Table 2). The groups did not differ in folate levels at any time point (Table 2).

**Table 2:** Vitamins and metabolites in infants aged 6 weeks, 4 months and 6 months according to nutrition^a^

		Duration of exclusive breastfeeding (Group)		*P* ^*b*^
		0–1 month (Formula fed)	>1 month (Breastfed)	
Number	At 6 weeks	32	48	
	At 4 months^c^	27	40	
	At 6 months^d^	26	40	
Serum cobalamin, pmol/L	At 6 weeks	372 (294, 444)	234 (158, 321)	<0.001
	At 4 months	476 (404, 573)	281 (224, 423)	<0.001
	At 6 months	497 (387, 622)	321 (198, 451)	<0.001
	*P* ^*e*^	<0.001	<0.001	
Serum folate, nmol/L	At 6 weeks	56.4 (30.6, 118,4)	27.2 (21.1, 119.9)	0.09
	At 4 months	61.4 (44.0, 84.5)	64.4 (41.8, 85.6)	0.96
	At 6 months	53.9 (34.2, 67.0)	50.5 (39.9, 62.5)	0.69
	*P* ^*e*^	0.48	0.02	
Plasma PLP, nmol/L	At 6 weeks	274 (201, 337)	79 (42, 132)	<0.001
	At 4 months	230 (155, 281)	135 (88, 161)	<0.001
	At 6 months	184 (123, 278)	122 (93, 162)	<0.001
	*P* ^*e*^	0.006	0.007	
Plasma riboflavin, nmol/L	At 6 weeks	62.2 (43.1, 84.1)	16.3 (13.8, 22.6)	<0.001
	At 4 months	36.3 (21.0, 47.2)	12.5 (9.8, 17.1)	<0.001
	At 6 months	33.5 (22.7, 49.5)	14.8 (10.6, 18.5)	<0.001
	*P* ^*e*^	0.001	0.02	
Plasma tHcy, *μ*mol/L	At 6 weeks	7.24 (5.91, 8.42)	7.44 (6.31, 9.07)	0.36
	At 4 months	5.90 (5.14, 7.26)	8.11 (6.40, 10.32)	<0.001
	At 6 months	5.38 (4.38, 6.96)	7.35 (5.78, 9.02)	0.001
	*P* ^*e*^	<0.001	0.50	
Plasma MMA, *μ*mol/L	At 6 weeks	0.61 (0.38, 1.14)	0.54 (0.28, 1.87)	0.59
	At 4 months	0.22 (0.20, 0.39)	0.50 (0.21, 1.32)	0.01
	At 6 months	0.19 (0.16, 0.36)	0.59 (0.33, 1.20)	<0.001
	*P* ^*e*^	<0.001	0.29	

^a^All values are medians, (IQR)

^b^Mann-Whitney U

^c^4 months: One blood sample missing 0–1 month, one missing for cobalamin and folate >1 month

^d^6 months: Four missing for PLP and riboflavin >1 month

^e^Friedman test

*PLP* Pyridoxal 5´-phosphate, *tHcy* total homocysteine, *MMA* Metylmalonic acid

**Fig. 1:**
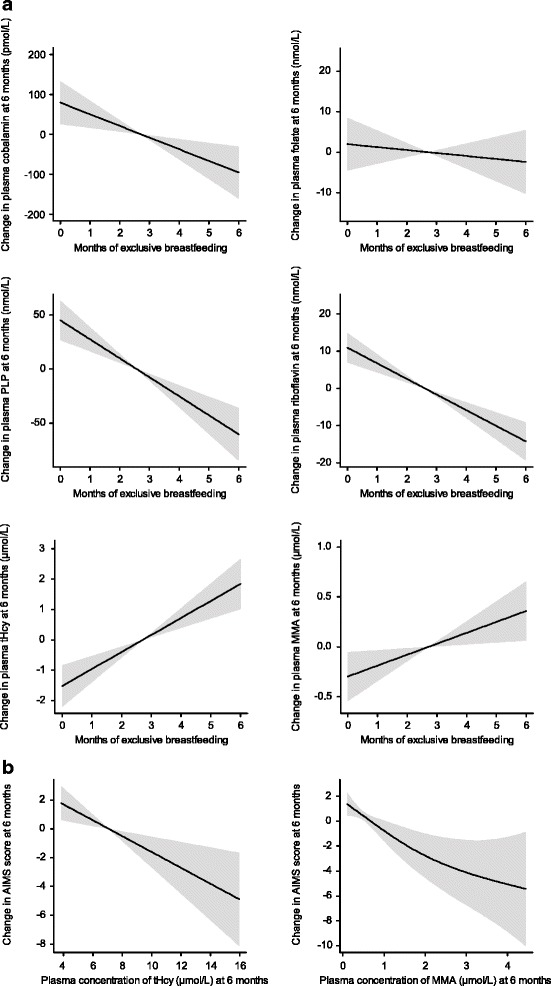
**a**. Dose-response relationship of cobalamin, folate, PLP, riboflavin, tHcy and MMA at 6 months with months of exclusive breastfeeding by Generalized additive models (GAM), adjusted for gender, infant weight at 6 months and iron and folate supplementation. The solid line shows the fitted model and the shaded areas indicate 95 % CIs. PLP, pyridoxal 5´phosphate; tHcy, total homocysteine; MMA, methylmalonic acid. **b**. Dose-response relationship of tHcy and MMA at 6 months with AIMS scores at 6 months by Generalized Additive Models (GAM), adjusted for gender, infant weight at 6 months and iron and folate supplementation. The solid line shows the fitted model and the shaded areas indicate 95 % CIs. tHcy, total homocysteine; MMA, methylmalonic acid

In a multiple linear regression model, which included gender, infant weight at 6 months, and iron and folate supplementation (i.e. for infants with BW ≤ 2500 g), the strongest determinant of infant B vitamin status at 6 months was duration (months) of exclusive breastfeeding (Table 3). B vitamin status at 6 months showed a linear, inverse relationship with duration (months) of exclusive breastfeeding, as shown by GAM (Fig. 1a).

**Table 3:** Determinants of B vitamin in infants aged 6 months (*n* = 66) by multiple linear regression^a^

Independent variables	Serum cobalamin		Serum folate		Plasma PLP		Plasma riboflavin		Plasma total homocysteine		Plasma methylmalonic acid	
	B	p	B	p	B	p	B	p	B	p	B	p
Gender (boys, girls)	25.65	0.67	-3.53	0.55	8.79	0.61	-0.32	0.93	-0.01	0.99	-0.03	0.90
Weight^b^	33.44	0.23	0.83	0.76	0.61	0.94	3.00	0.09	-0.07	0.80	-0.15	0.15
Exclusive breastfeeding^c^	-44.32	0.001	-0.76	0.53	-17.53	<0.001	-4.16	<0.001	0.55	<0.001	0.12	0.008

^a^The regression model contains folic acid and iron supplementations as independent variables, in addition to the parameters listed in the table

^b^Infant weight at 6 months, quartiles

^c^Exclusive breastfeeding, number of months with exclusive breastfeeding from birth to 6 months

*PLP* Pyridoxal 5´- phosphate, B: regression coefficient

When comparing infants with BW ≤ 2500 g and BW 2501-3000 g, we observed no differences in B vitamin levels and the metabolic markers at 4 or 6 months (*p* > 0.13) except for folate at 6 weeks and 4 months, which was higher in infants BW ≤ 2500 g, who had been supplemented with folic acid (*p* < 0.001).

### Neurodevelopment in relation to breastfeeding practice and B vitamin status

AIMS data were available for 61 of the 66 (92 %) infants at 6 months. Of the 5 infants with missing data, 3 came from the formula fed and 2 from the breastfed group. The formula fed infants had a significantly higher median AIMS score than the breastfed infants (Table 1).

In the breastfed group 25/38 (66 %) infants scored below the 50^th^ percentile and 8/38 (21 %) below the 10^th^ percentile, i.e. classified as possibly delayed motor development, compared to 9/23 (39 %, *p* = 0.04) and 3/23 (13 %, *p* = 0.43) in the formula fed group.

Duration of exclusive breastfeeding was a significant negative predictor of AIMS score in a multiple linear regression model adjusted for gender, SGA, infant weight at 6 months, maternal education and folate and iron supplementations (B = -0.5; (95 % CI; -0.9 - -0.03, *p* = 0.04) per month of exclusive breastfeeding). The dose-response reduction in AIMS score with increasing levels of tHcy and MMA is visualized by GAM curves in Fig. 1b.

ASQ data were available for 61 of the 66 (92 %) infants at 6 months (missing data for 2 infants in the formula fed and for 3 infants in the breastfed group). The breastfed infants had a significantly lower median gross motor score (*p* = 0.01) and the median fine motor score showed a similar trend (*p* = 0.06). No significant differences were observed for communication, personal-social functioning and problem solving skills (*p* > 0.09) (Table 1).

### Cobalamin intervention

At 6 months, 36 (45 %) of the 66 infants had plasma tHcy > 6.5 μmol/L and were invited to participate in the intervention study. Of these, 32 infants accepted and were included (cobalamin group, *n* = 16 and placebo group, *n* = 16). All, but one infant (from the placebo group), came back for assessment after one month.

At inclusion, there were no significant differences between the cobalamin and the placebo group for infant characteristics (growth parameters at birth and 6 months, GA, SGA and twin status, use of vitamins and iron, AIMS score and ASQ scores) or maternal characteristics (age, pre pregnancy BMI and parity) (*p* > 0.06). There were however, more girls in the cobalamin group (11/16) than in the placebo group (4/16) (p = 0.01) and infants in the cobalamin group were exclusively breastfed for a longer period (median 5 months (IQR 3, 6)) compared to the placebo group (3 months (0, 5), p = 0.03). This was reflected in significantly higher tHcy levels (median 9.57 μmol/L (IQR 7.62, 11.61)) in the cobalamin group compared to the placebo group (7.72 μmol/L (6.91, 8.33), p = 0.02) at inclusion. No other significant differences in metabolic parameters were seen (*p* > 0.16).

The observed changes in cobalamin, tHcy, and MMA levels from inclusion to follow-up were significantly greater in the cobalamin compared to the placebo group (Table 4), while no significant differences between the two groups were observed for the other vitamins. AIMS and ASQ scores increased in both groups from inclusion at age 6 months to follow-up at age 7 months as expected; however, the median increase in scores for AIMS and for ASQ gross motor function were significantly higher for the cobalamin group than the placebo group (Table 4). There were no significant differences between the groups for fine motor score, communication, personal-social functioning or problem solving skills (*p* > 0.4). No adverse effects from the cobalamin injections were reported.

**Table 4:** Change in biochemical status and clinical parameters according to cobalamin intervention at 6 months and follow-up at 7 months

	Trial Groups (tHcy: 6.73–15.96)		*P* value
Change in variables	Cobalamin Group	Placebo Group	
Number	16/16	16/15	
Serum cobalamin, pmol/L, (median (IQR)), %change	707 (422, 904), 254 %	33 (-17, 74), 10 %	<0.001^a^
Plasma total homocysteine, μmol/L, (median (IQR)), %change	-5.85 (-7.48, -4.37), -61 %	-1.02 (-1.81, -0.23), -13 %	<0.001^a^
Plasma methylmalonic acid, μmol/L, (median (IQR)), %change	-0.88 (-2.01, -0.12), -113 %	-0.07 (-0.33, 0.29), -14 %	0.001^a^
Serum folate, nmol/L, (median (IQR)), %change	-16.1 (-30.4, -2.5), -37 %	-14.0 (-16.8, -2.9), -29 %	<0.44^a^
Plasma PLP, μmol/L, (median (IQR)), %change	12 (-24, 38), 9 %	0 (-22, 61), 0 %	<0.98^a^
Plasma riboflavin, μmol/L, (median (IQR)), %change	0.3 (-4.8, 2.7), 2 %	3.7 (-3.5, 8.4), 3 %	<0.32^a^
AIMS score, (median (IQR)), %change	7.0 (5.3, 9.8), 36 %	5.0 (4.0, 7.0), 23 %	0.02^a^
ASQ; Gross motor score (median (IQR)), %change^c^	12.5 (10.0, 16.3), 42 %	10.0 (-1.3, 10.0), 29 %	0.03^a^
Weight, gram, (mean (SD)), %change	532 (230), 8 %	377 (257), 6 %	0.09^b^
Length, cm, (mean (SD)), %change	2.0 (1.3), 3 %	1.8 (1.1), 3 %	0.81^b^
Head circumference, cm, (mean (SD)), %change	0.8 (0.7), 2 %	0.8 (0.4), 2 %	0.89^b^

^a^Medians were compared by Mann-Whitney *U* test

^b^Means were compared by Student’s *t*-test

^c^Missing data for 2 infants in the Cobalamin group and 4 infants in the Placebo group

*PLP* Pyridoxal 5´- phosphate, *AIMS* Alberta Infant Motor Scale, *ASQ* Ages and Stages Questionnaires

## Discussion

In the present study of infants with BW between 2000-3000 g, those who were mainly formula fed from birth had significantly higher levels of cobalamin, PLP and riboflavin and lower levels of the metabolic markers, tHcy and MMA, and a better gross motor development at 6 months compared to infants who were exclusively breastfed for more than 1 month, despite the fact that the formula fed group had more twins and lower maternal educational level, factors known to be negatively associated with neurodevelopment [43, 44]. Furthermore, vitamin status, as well as gross motor function, was negatively and linearly associated with duration of exclusive breastfeeding when adjusted for possible confounders.

In infants with biochemical signs of mild cobalamin deficiency at 6 months, cobalamin treatment resulted in significant improvement in cobalamin status and motor function. These results indicate that the observed impairment in motor function associated with long-term exclusive breastfeeding, may be due to cobalamin deficiency.

### Study design and limitations

The first part of this study was observational, known to have its limitations. However, data were collected prospectively, the participation rate was high throughout the study and there were no significant differences in infant or maternal characteristics between the two groups that could explain the differences in clinical outcome.

Evaluation of motor development, a major developmental function in early infancy [36, 45] is challenging [46]. Infants develop discontinuously, and the age of achieving gross motor milestones varies substantially among healthy term infants [47]. The AIMS test is considered to be among the most reliable tests for assessing gross motor function [36, 45] and ASQ is a validated screening tool with high sensitivity and specificity to detect children with developmental delay [38]. It was a weakness of the study that the examiner was not blinded to the nutrition of the infants when the infants were first assessed at 6 months, however, as all AIMS scores were revised based on the videotape, without access to clinical data, after the study was completed, potential confounding was minimized. In the intervention study, both the parents and the examiner were blinded to the intervention when assessing the infants 1 month after randomization.

The intervention study included 86 % of eligible infants with cobalamin deficiency at 6 months. Apart from differences in gender and period of exclusive breastfeeding, similar characteristics of the cobalamin and placebo groups suggest that the randomization was appropriate. The given dose of 400 μg hydroxycobalamin represents approximately twice the total amount of cobalamin considered necessary for the first year of life, based on an Adequate Intake (AI) for cobalamin [48]. This dosage has been proven to improve cobalamin status and enhance motor development in young infants [22].

### B vitamin status and psychomotor development

Gross motor function is a good marker of neurodevelopment in early infancy [45, 49], and is known to be related to micronutrient status [25, 26]. We have earlier demonstrated in a randomized, double blind intervention study that cobalamin supplementation not only improves biochemical measures of cobalamin status, but also motor development and gastrointestinal symptoms in moderately cobalamin-deficient infants, an observation that emphasizes the importance of an adequate cobalamin status for normal neurodevelopment [22]. In the present study, formula fed infants had significantly better B vitamin status and higher median AIMS and ASQ scores compared to the breastfed infants. We cannot exclude that nutrients other than B vitamins, may at least partially, have contributed to the observed differences in clinical outcome. Our study population consisted of infants born with a suboptimal BW, and one may assume that they had a higher risk of micronutrient deficiency compared to infants born AGA close to term. Motor development was, however, not related to BW or AGA vs. SGA status. Motor development is influenced by several factors, like GA, BW, neonatal health and genetic, cultural and parental sociodemographic factors [43, 50]. After adjusting for such factors, the associations between gross motor function and duration of exclusive breastfeeding remained, suggesting that at least cobalamin status had a significant effect on gross motor function. The intervention study confirmed this notion, as our results indicate that the observed impairment in motor function associated with long-term exclusive breastfeeding is corrected by cobalamin supplementation.

### Prolonged exclusive breastfeeding and adequate micronutrient status

With the exception of vitamin D and K, which are supplemented, the World Health Organization (WHO) considers breast milk to be a complete food for the term infant for the first 6 months of life, a period of rapid growth and development [51]. Low BW (<2500 g) is a recognized risk factor for multiple micronutrient deficiencies, although supplementation with only iron and folic acid are commonly recommended [52–54].

We observed a higher MMA level, despite a similar cobalamin level, indicative of inadequate intracellular cobalamin status, in the breastfeeding compared to the formula feeding mothers at 6 months. Cobalamin levels in milk correlate with maternal plasma levels [55] and falls progressively during the lactional period [12, 19]. The estimated cobalamin intake from breastmilk has been reported to be maximal at 12 weeks, and reduced by 50 % at 24 weeks [56], which may not be satisfactory given the crucial role for cobalamin in neurodevelopment [20].

The present study suggests that prolonged exclusive breastfeeding may not sustain sufficient B vitamin status, not only for those with a low BW, but also for infants with a BW in the range 2500–3000 g. Although all B vitamins, except for folate, were lower in breastfed infants already from 6 weeks, the metabolic markers were significantly higher from 4 months, suggesting an intracellular B vitamin deficency in exclusively breastfed infants at this age. As B vitamins are important for development, these data suggest that introduction of solid animal food should start from age 3–4 months.

## Conclusion

In this study, duration of exclusive breastfeeding was associated with lower B vitamin status and poorer gross motor development at 6 months in infants with BW 2000-3000 g. In infants with biochemical signs of mild cobalamin deficiency at 6 months, cobalamin treatment resulted in significant improvement in cobalamin status and motor function. These results indicate that the observed impairment in motor function associated with long-term exclusive breastfeeding, may be due to cobalamin deficiency. In order to obtain an adequate cobalamin status to ensure normal neurodevelopment, we suggest that introduction of solid animal food should start from age 4 months in infants with a subnormal BW.

## Abbreviations

AGA: Appropriate weight for Gestational Age
AIMS: Alberta Infant Motor Scale
ASQ: Ages and Stages Questionnaire
BW: Birth Weight
G: Grams
GA: Gestational Age
GAM: Generalized Additive Models
tHcy: Plasma levels of total plasma homocysteine
IQR: Interquartile Range
MMA: Methylmalonic Acid
PLP: Pyridoxal 5´-phosphate
SD: Standard Deviation
SGA: Small for Gestational Age
